# Development and internal validation of a nomogram for estimating concurrent superficial vein thrombosis at presentation in patients with lower extremity varicose veins

**DOI:** 10.3389/fmed.2026.1889714

**Published:** 2026-10-02

**Authors:** Lei Jiao, Zhen Zhou, Hongsheng Yin

**Affiliations:** 1 School of Information and Control Engineering, China University of Mining and Technology, Xuzhou, China; 2 Department of Information, Xuzhou Mining Group General Hospital, Xuzhou, China

**Keywords:** feature selection, logistic regression, lower extremity varicose veins, nomogram, superficial vein thrombosis

## Abstract

**Background:**

Superficial vein thrombosis (SVT) is a clinically relevant complication of lower extremity varicose veins. This study aimed to develop and internally validate a parsimonious nomogram for estimating the probability of concurrent SVT at presentation.

**Methods:**

This single-center retrospective study included 423 patients with lower extremity varicose veins, including 91 with SVT. Twenty-four candidate variables were evaluated using LASSO regression, the Boruta algorithm, and a genetic algorithm for feature selection. Variables retained by all three methods were entered into multivariable logistic regression to construct the full model, and a reduced model excluding fibrinogen (FIB) was assessed for parsimony. Model performance was compared with the Caprini score and internally validated using 1,000 bootstrap resamples.

**Results:**

Age, interleukin-6, C-reactive protein, D-dimer, and FIB were identified as consensus-selected variables. The full model had an AUC of 0.844 (95% CI, 0.797–0.891). Removing FIB produced minimal changes in discrimination (*Δ*AUC = 0.007; 95% CI, −0.004 to 0.017; *P* = 0.224), calibration, Brier score, or decision curves. The four-variable model was therefore selected for nomogram presentation. Its AUC was 0.837 (95% CI, 0.790–0.884), compared with 0.722 (95% CI, 0.663–0.782) for the Caprini score (*Δ*AUC = 0.115; 95% CI, 0.040–0.190; *P* = 0.003). The optimism-corrected AUC was 0.837. Adding the Caprini score to the full model numerically increased the AUC from 0.844 to 0.871 (*Δ*AUC = 0.027; 95% CI, 0.000–0.055; *P* = 0.054).

**Conclusion:**

A nomogram based on age, interleukin-6, C-reactive protein, and D-dimer showed good internally validated performance for estimating the probability of concurrent SVT at presentation. Because validation was limited to bootstrap resampling within a single-center cohort, independent external validation is required before clinical application.

## Introduction

Lower extremity varicose veins are a common chronic venous disease in clinical practice, primarily characterized pathologically by venous hypertension and the tortuous dilation of superficial veins (1, 2). As the disease progresses, a subset of patients may develop superficial venous thrombosis (SVT) (3). SVT not only causes acute local erythema, swelling, and pain, severely impairing patients' quality of life, but the thrombus can also extend to the saphenofemoral junction or invade the deep venous system via perforating veins. This consequently increases the risk of secondary deep vein thrombosis (DVT) and even pulmonary embolism (PE) (4, 5). Therefore, timely identification of concurrent SVT among patients with lower extremity varicose veins is important for clinical assessment and management.

Currently, clinical practice frequently relies on standardized tools such as the Caprini score for the risk assessment of venous thromboembolism (VTE) (6, 7). However, the Caprini score was originally designed for broad-spectrum thrombosis prophylaxis in general hospitalized and perioperative populations; its assessment items cover a wide range and are not specifically optimized for the unique microenvironmental characteristics of lower extremity varicose veins (8). Furthermore, traditional assessment scales largely depend on structured clinical histories and physical examinations, failing to adequately integrate objective biochemical markers that reflect endothelial injury, inflammatory activation, and hypercoagulable states within the body. This lack of deep biological information limits, to some extent, the discriminative performance and disease specificity of such tools for identifying SVT secondary to varicose veins.

The pathological mechanism of SVT involves a complex interplay of venous stasis, vascular endothelial injury, and abnormal systemic coagulation mechanisms (9, 10). Recent studies indicate that the activation of the “inflammation-coagulation axis” plays a fundamental role in the evolution of thrombotic diseases, and various systemic inflammatory markers alongside coagulation and fibrinolysis-related indices have been proven to be associated with endothelial dysfunction and prothrombotic states (11, 12). However, when introducing multidimensional clinical histories and serological candidate indicators, traditional statistical screening methods struggle to effectively handle collinearity and redundant information among high-dimensional data (13). In contrast, machine learning algorithms can integrate diverse mathematical mechanisms—such as regularization penalization, feature importance evaluation, and heuristic global search—to effectively overcome the collinearity challenges of high-dimensional data. Because different feature-selection methods rely on distinct selection criteria, their combined use can provide complementary information (14). In this study, the intersection of LASSO, Boruta, and GA results was used as a conservative consensus rule to derive a parsimonious set of variables supported across all three methods.

Against this background, this study aimed to integrate demographic characteristics, clinical signs, and inflammatory and coagulation biomarkers in patients with lower extremity varicose veins, using a combined machine-learning feature-selection strategy to construct a nomogram for individualized estimation of the probability of concurrent SVT at presentation. The model was also compared with the traditional Caprini score to evaluate its performance in identifying concurrent SVT within this patient population. The present study was intended as model development and internal validation rather than establishment of a tool for immediate clinical implementation.

## Methods

### Study population

This single-center retrospective observational study consecutively screened all patients with lower-extremity varicose veins who were assessed for eligibility at Xuzhou Mining Group General Hospital between January 2021 and December 2024. All eligible patients identified during the study period were considered for inclusion according to the predefined inclusion and exclusion criteria. The inclusion criteria were age ≥18 years, a definitive diagnosis of lower extremity varicose veins, and definitive determination of concurrent SVT status. The study was conducted in accordance with the Declaration of Helsinki and approved by the Ethics Committee of Xuzhou Mining Group General Hospital (Approval No. 2025110701). The requirement for informed consent was waived because of the retrospective design and use of de-identified data.

Among 443 patients meeting the inclusion criteria, 20 were excluded: 3 with concomitant deep vein thrombosis or pulmonary embolism, 2 with a previous history of venous thrombosis, 4 with acute infection, malignancy, hematological disease, autoimmune disease, or established coagulopathy, 1 who was pregnant or lactating, 2 with recent major surgery, severe trauma, or treatments substantially affecting inflammatory or coagulation status, and 8 with incomplete interleukin-6 (IL-6), C-reactive protein (CRP), D-dimer, and/or fibrinogen (FIB) measurements. The final complete-case cohort comprised 423 patients, including 91 with SVT and 332 without SVT. For patients with repeated visits or hospitalizations, only the first eligible record was retained (Figure 1).

**Figure 1:**
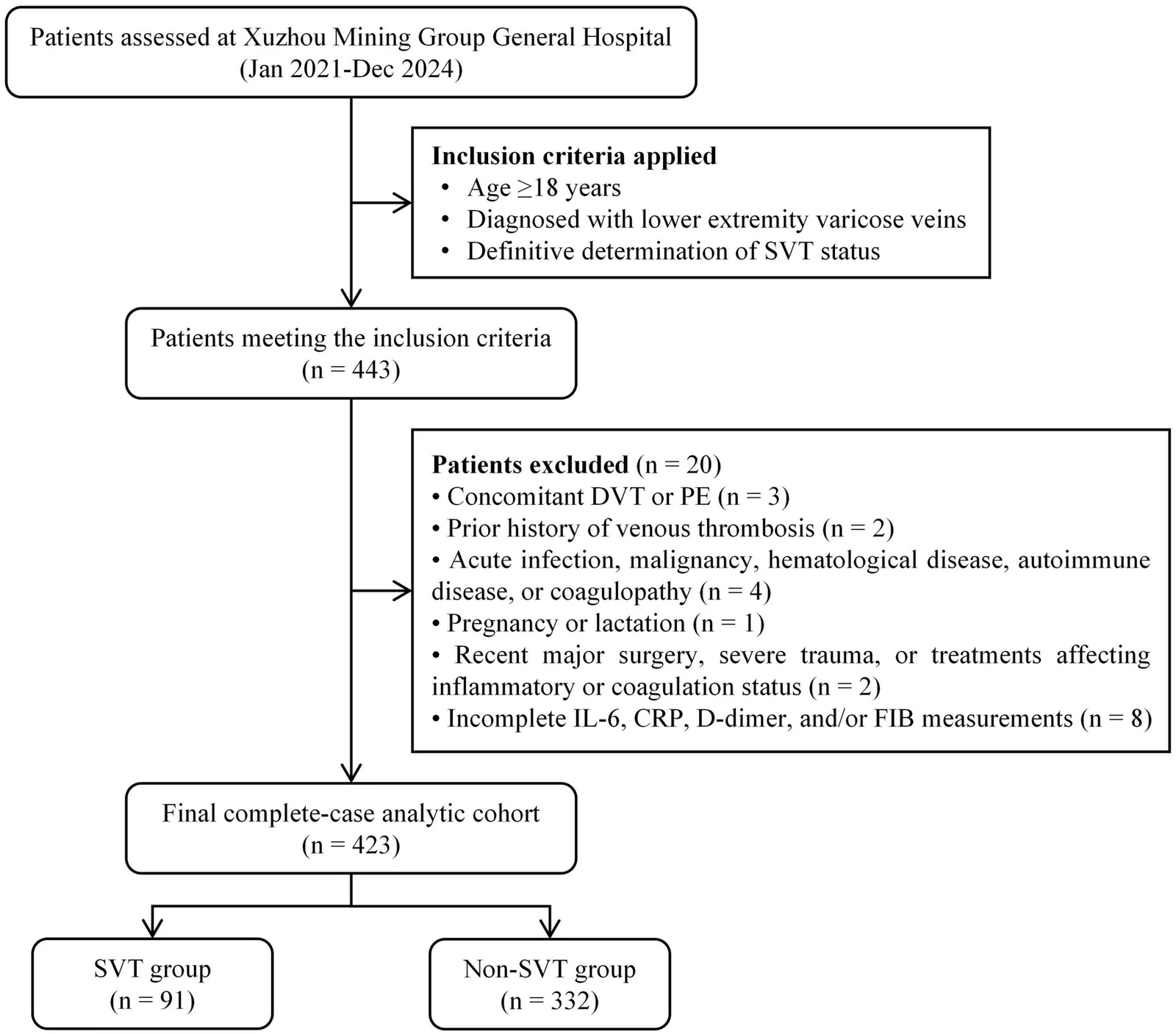
Flowchart of patient selection and analytic cohort construction. Among 443 screened patients with lower extremity varicose veins, 423 patients were included in the final analytic cohort, comprising 91 patients with concurrent SVT and 332 without concurrent SVT. SVT, superficial vein thrombosis; DVT, deep vein thrombosis; PE, pulmonary embolism; IL-6, interleukin-6; CRP, C-reactive protein; FIB, fibrinogen.

### Outcome ascertainment

The primary outcome was the presence of concurrent SVT in patients with lower extremity varicose veins. SVT was diagnosed on the basis of clinical manifestations combined with color Doppler ultrasound findings. Clinical manifestations included acute erythema, swelling, warmth, and pain along the course of a varicose superficial vein, with or without cord-like induration or a palpable local mass. Diagnostic ultrasound findings included abnormal intraluminal echoes, partial or complete incompressibility of the superficial vein, and reduced or absent local blood-flow signals.

### Clinical data collection

Baseline data collected within 24 h of the initial visit or admission included age, sex, body mass index, smoking and drinking status, hypertension, diabetes, coronary heart disease, cerebral infarction, antiplatelet, lipid-lowering and venoactive drug use, affected-limb location, Clinical-Etiology-Anatomy-Pathophysiology (CEAP) classification, leg swelling, white blood cell count, red blood cell count, platelet count, IL-6, CRP, D-dimer, prothrombin time, activated partial thromboplastin time, FIB, and the Caprini score. The Caprini score was prespecified as the traditional thrombosis-risk comparator. Categorical variables were coded according to their recorded categories. CEAP clinical classes were defined according to the updated CEAP classification system. For analysis, patients were categorized into C2-C3 and C4-C6 groups according to the clinical manifestations of chronic venous disease, representing varicose veins/edema and advanced skin or ulcerative changes, respectively (4, 15).

Recent immobilization and quantitative measures of venous stasis or mechanical severity, including great saphenous vein diameter, saphenofemoral junction reflux duration, and standardized hemodynamic measurements, were not consistently documented in the retrospective records and were therefore not included in the candidate variable pool.

### Laboratory assessment

Fasting venous blood samples were collected within 24 h of the initial visit or admission and analyzed using standardized procedures on the same hospital laboratory platform. The first available measurement of each laboratory variable was used; clinically implausible values were verified against the original records and corrected or excluded only when a recording or measurement error was confirmed. Because biomarker measurement and SVT ascertainment occurred during the same presentation period, these laboratory variables were treated as contemporaneous characteristics rather than antecedent risk factors for SVT.

### Missing-data assessment

Missing observations were limited to IL-6 (*n* = 7, 1.58%), CRP (*n* = 4, 0.90%), D-dimer (*n* = 3, 0.68%), and FIB (*n* = 3, 0.68%). Eight patients had at least one missing biomarker measurement, with some missing more than one biomarker. No other candidate variable or Caprini score value was missing. Because missingness for each affected variable was below 5%, complete-case analysis was performed without multiple imputation.

To assess potential selection imbalance associated with complete-case exclusion, available baseline characteristics were compared between the 423 included patients and the 8 patients excluded solely because of missing biomarker data. Between-group differences were evaluated using the statistical tests described below and absolute standardized mean differences. Given the small number of excluded patients, both *P* values and standardized mean differences were interpreted cautiously.

### Feature selection and model development

All 24 prespecified demographic, clinical, disease-related, medication-related, and laboratory variables were included in the candidate variable pool without univariable *P*-value-based preselection. The Caprini score was prespecified solely as a comparator and excluded before feature selection. A consensus strategy integrating least absolute shrinkage and selection operator (LASSO) regression, the Boruta algorithm, and a genetic algorithm (GA) was used. Variables retained by all three methods were defined as consensus-selected variables. The intersection rule was chosen as a conservative modeling strategy to prioritize parsimony and cross-method agreement and to reduce reliance on variables selected by only one method; it was not intended to define an objectively established set of variables. The use of three complementary methods was intended to reduce dependence on any single selection criterion rather than to increase model complexity. The consensus-selected variables were entered simultaneously into a multivariable logistic regression model to construct the full data-driven model (Model 3). To evaluate the incremental contribution of FIB and model parsimony, a reduced model excluding FIB was additionally evaluated as Model 2. The Caprini score alone constituted Model 1, while Model 4 combined Model 3 with the Caprini score. LASSO was implemented using glmnet version 4.1.10 with family = “binomial”, alpha = 1, and internal standardization; lambda.min was selected by 10-fold cross-validation using binomial deviance. Boruta was implemented using Boruta version 9.0.0 with maxRuns = 500 and pValue = 0.01; tentative variables were resolved using TentativeRoughFix. The GA was implemented using GA version 3.2.5 with binary encoding, population size 100, maximum 40 generations, crossover probability 0.80, mutation probability 0.10, elitism 5, and termination after 10 generations without fitness improvement. For each GA-generated subset, logistic regression was evaluated using stratified five-fold cross-validation, with the mean AUC used as the fitness value. Because the GA is stochastic, selection stability was additionally evaluated by repeating the complete GA procedure 100 times using different random seeds while keeping all tuning parameters unchanged. Variable-level selection frequencies were calculated across the 100 independent runs. Continuous variables were modeled on their original scales without categorization or logarithmic transformation; standardization was used only during LASSO selection. Their functional forms were subsequently evaluated using multivariable restricted cubic spline analyses as described in the Statistical analysis section. Spearman correlation coefficients and variance inflation factors were used to assess pairwise correlations and multicollinearity, respectively, with VIF <5 indicating no substantial multicollinearity.

### Internal validation and optimism correction

Internal validation used 1,000 bootstrap resamples. Within each resample, the complete feature-selection pipeline, including LASSO, Boruta, GA optimization, and consensus selection, was repeated *de novo* for the full data-driven Model 3. The resulting consensus-selected variables were used to reconstruct and fit Model 3 within that bootstrap sample, and the Caprini score was additionally incorporated to construct Model 4. Model 1 was refitted as the fixed Caprini-only comparator, whereas Model 2 was refitted according to its fixed four-variable specification without repeating feature selection. Thus, the complete feature-selection pipeline was repeated *de novo* for Model 3 in each bootstrap resample; Model 4 was then constructed by adding the Caprini score to the bootstrap-specific Model 3, whereas Models 1 and 2 were refitted according to their fixed specifications without repeating feature selection. To assess feature-selection stability across resampled datasets, the variables retained by the three-method consensus were recorded in each of the 1,000 bootstrap resamples, and variable-level selection frequencies were calculated for all 24 candidate variables. Each bootstrap-developed model was subsequently applied to the original cohort without further feature selection or model refitting. AUC optimism was calculated as the difference between the performance of each bootstrap-developed model in the bootstrap sample and its performance when applied to the original cohort. Mean optimism was subtracted from the apparent AUC, and calibration intercepts, calibration slopes, and Brier scores were corrected using the same framework. A prespecified random seed [set.seed(1234)] was set once before resampling, which continued until 1,000 valid iterations were obtained.

### Multiple-imputation sensitivity analysis

To assess the robustness of the findings to complete-case exclusion, a sensitivity analysis was performed in all clinically eligible patients using multiple imputation by chained equations. Missing values for IL-6, CRP, D-dimer, and FIB were imputed using predictive mean matching, with 20 imputed datasets, 20 iterations, and five donors. The SVT outcome and all available baseline variables were included as predictors in the imputation models, while the outcome itself was not imputed. The predefined specifications of Models 1–4 were refitted in each imputed dataset without repeating feature selection. Model coefficients were combined using Rubin's rules, and model performance was reassessed across the imputed datasets.

### Statistical analysis

Normally distributed continuous variables were expressed as mea*n* ± standard deviation and compared using the independent-samples t test; skewed variables were expressed as median and interquartile range and compared using the Mann–Whitney U test. Categorical variables were presented as frequencies and percentages and compared using the *χ*^2^ test or Fisher's exact test, as appropriate. Four models were evaluated: the Caprini score alone (Model 1); age, IL-6, CRP, and D-dimer (Model 2); age, IL-6, CRP, D-dimer, and FIB (Model 3); and Model 3 plus the Caprini score (Model 4). The functional forms of the continuous variables retained for model development were evaluated using multivariable restricted cubic spline logistic regression with three knots. Age, IL-6, CRP, D-dimer, and FIB were each examined within the corresponding multivariable model framework, with the remaining variables retained as adjustment covariates. The median value of each variable was used as the reference value (odds ratio=1.0). Overall associations and departures from linearity on the log-odds scale were assessed using the corresponding spline tests, with *P* for nonlinearity <0.05 indicating evidence of a nonlinear relationship. Variables without evidence of nonlinearity were retained as linear terms on their original scales in the final logistic regression models. Discrimination was assessed using ROC curves and AUCs with 95% confidence intervals, and correlated AUCs were compared using DeLong's test. Calibration was assessed using calibration curves, calibration intercepts, calibration slopes, and Brier scores, while clinical utility was assessed primarily using decision curve analysis. The threshold probabilities used in decision curve analysis were analytical thresholds for evaluating net benefit and were not prespecified or validated clinical decision cut-offs. Pairwise comparisons included Model 2 versus Model 1, Model 3 versus Model 1, Model 3 versus Model 2, and Model 4 versus Model 3. For each comparison, continuous NRI, IDI, and the absolute AUC difference, together with their 95% confidence intervals, were estimated using 500 paired bootstrap resamples. Correlated AUCs were additionally compared using DeLong's test. Within each pairwise comparison, the two models were evaluated using identical bootstrap resamples. Model 2 versus Model 3 assessed the incremental contribution of FIB, whereas Model 3 versus Model 4 assessed the incremental contribution of the Caprini score. Continuous NRI was treated as exploratory because it captures probability changes regardless of magnitude. Categorical NRI was not calculated because no prespecified, externally validated probability categories for concurrent SVT were available. Reclassification metrics were considered secondary analyses and were not used as the primary evidence of clinical utility; clinical net benefit was assessed primarily using decision curve analysis. For the Model 3 versus Model 4 comparison, the AUC difference, its 95% confidence interval, and the corresponding DeLong *P* value were reported without inferring additional discriminative or clinical value. Internal validation and optimism correction were performed as described above. Analyses used *R* version 4.5.1, with two-sided *P* < 0.05 denoting statistical significance.

## Results

### Baseline clinical and laboratory characteristics

A total of 423 patients were included in the complete-case analytic cohort, comprising 332 patients without SVT and 91 patients with SVT. Compared with the non-SVT group, patients with SVT were older, had a higher BMI, and were more frequently male. Hypertension, diabetes, bilateral lower-extremity involvement, and leg swelling were also more common in the SVT group, which also had a higher proportion of patients with severe CEAP classification. Patients with SVT had higher WBC and PLT counts, higher levels of IL-6, CRP, D-dimer, and FIB, longer PT and APTT, and a higher mean Caprini score. Lipid-lowering and venoactive drug use was also more frequent in the SVT group. No statistically significant differences were observed in smoking, drinking, coronary heart disease, cerebral infarction, antiplatelet drug use, or RBC count (Table 1).

**Table 1:** Baseline characteristics of patients according to concurrent SVT status.

Variables	Total (*n* = 423)	Non-SVT group (*n* = 332)	SVT group (*n* = 91)	*P*
Age, years	59.16 ± 11.56	58.15 ± 11.71	62.84 ± 10.23	<0.001
Male, n(%)	172 (40.66)	123 (37.05)	49 (53.85)	0.004
BMI, kg/m^2^	25.37 ± 3.41	25.14 ± 3.29	26.21 ± 3.71	0.008
Smoking, n(%)	125 (29.55)	94 (28.31)	31 (34.07)	0.287
Drinking, n(%)	94 (22.22)	69 (20.78)	25 (27.47)	0.174
Coronary heart disease, n(%)	50 (11.82)	36 (10.84)	14 (15.38)	0.235
Hypertension, n(%)	161 (38.06)	115 (34.64)	46 (50.55)	0.006
Diabetes, n(%)	79 (18.68)	50 (15.06)	29 (31.87)	<0.001
Cerebral infarction, n(%)	34 (8.04)	28 (8.43)	6 (6.59)	0.567
Antiplatelet drugs, n(%)	85 (20.09)	61 (18.37)	24 (26.37)	0.092
Lipid-lowering drugs, n(%)	120 (28.37)	85 (25.60)	35 (38.46)	0.016
Venoactive drugs, n(%)	192 (45.39)	139 (41.87)	53 (58.24)	0.005
Leg swelling, n(%)	176 (41.61)	125 (37.65)	51 (56.04)	0.002
CEAP classification, n(%)				0.025
C2-C3	270 (63.83)	221 (66.57)	49 (53.85)	
C4-C6	153 (36.17)	111 (33.43)	42 (46.15)	
Affected limb, n(%)				0.023
Unilateral	342 (80.85)	276 (83.13)	66 (72.53)	
Bilateral	81 (19.15)	56 (16.87)	25 (27.47)	
WBC, ×10^9^/L	7.33 ± 1.50	7.11 ± 1.41	8.12 ± 1.56	<0.001
RBC, ×10^12^/L	4.50 ± 0.47	4.51 ± 0.46	4.47 ± 0.47	0.495
PLT, ×10^9^/L	235.29 ± 48.17	231.11 ± 46.44	250.56 ± 51.46	<0.001
IL-6, pg/mL	9.55 ± 4.73	8.21 ± 3.51	14.42 ± 5.39	<0.001
CRP, mg/L	7.85 ± 5.30	6.30 ± 3.39	13.49 ± 6.95	<0.001
D-dimer, mg/L	1.01 ± 0.94	0.75 ± 0.50	1.94 ± 1.45	<0.001
PT, s	11.88 ± 1.14	11.80 ± 1.09	12.15 ± 1.29	0.011
APTT, s	30.93 ± 3.61	30.68 ± 3.42	31.80 ± 4.13	0.019
FIB, g/L	3.39 ± 0.78	3.26 ± 0.70	3.90 ± 0.84	<0.001
Caprini score	5.38 ± 1.81	4.90 ± 1.58	7.10 ± 1.56	<0.001

Continuous variables are presented as mea*n* ± standard deviation, and categorical variables as number (percentage), unless otherwise indicated.

SVT, superficial vein thrombosis; CEAP, Clinical-Etiology-Anatomy-Pathophysiology; BMI, body mass index; WBC, white blood cell count; RBC, red blood cell count; PLT, platelet count; IL-6, interleukin-6; CRP, C-reactive protein; PT, prothrombin time; APTT, activated partial thromboplastin time; FIB, fibrinogen.

Among the 431 patients remaining after application of the clinical exclusion criteria, 423 were retained in the complete-case analytic cohort and 8 were excluded because of incomplete biomarker measurements. None of the calculable comparisons of available baseline characteristics between the included and excluded patients reached statistical significance. However, appreciable absolute standardized mean differences were observed for affected-limb distribution, diabetes, antiplatelet drug use, PT, drinking status, CEAP classification, coronary heart disease, age, and PLT count. Potential selection imbalance associated with complete-case exclusion therefore could not be excluded. Given the small number of excluded patients, both the hypothesis-test results and standardized differences were interpreted cautiously (Supplementary Table S1).

### Consensus feature selection and multicollinearity assessment

All 24 prespecified candidate variables were evaluated using LASSO regression, the Boruta algorithm, and a genetic algorithm (GA), without univariable significance-based preselection. The Caprini score was excluded from the candidate variable pool and evaluated separately as a comparator. In the LASSO analysis, 10-fold cross-validation was used to determine the optimal penalty parameter, and variables with nonzero coefficients at lambda.min were retained (Figures 2A,B). The Boruta algorithm assessed the importance of the original variables relative to shadow variables and classified variables as confirmed or rejected (Figure 2C). The GA iteratively optimized candidate variable subsets, with the average, median, and maximum fitness scores gradually stabilizing over successive generations (Figure 2D).

**Figure 2:**
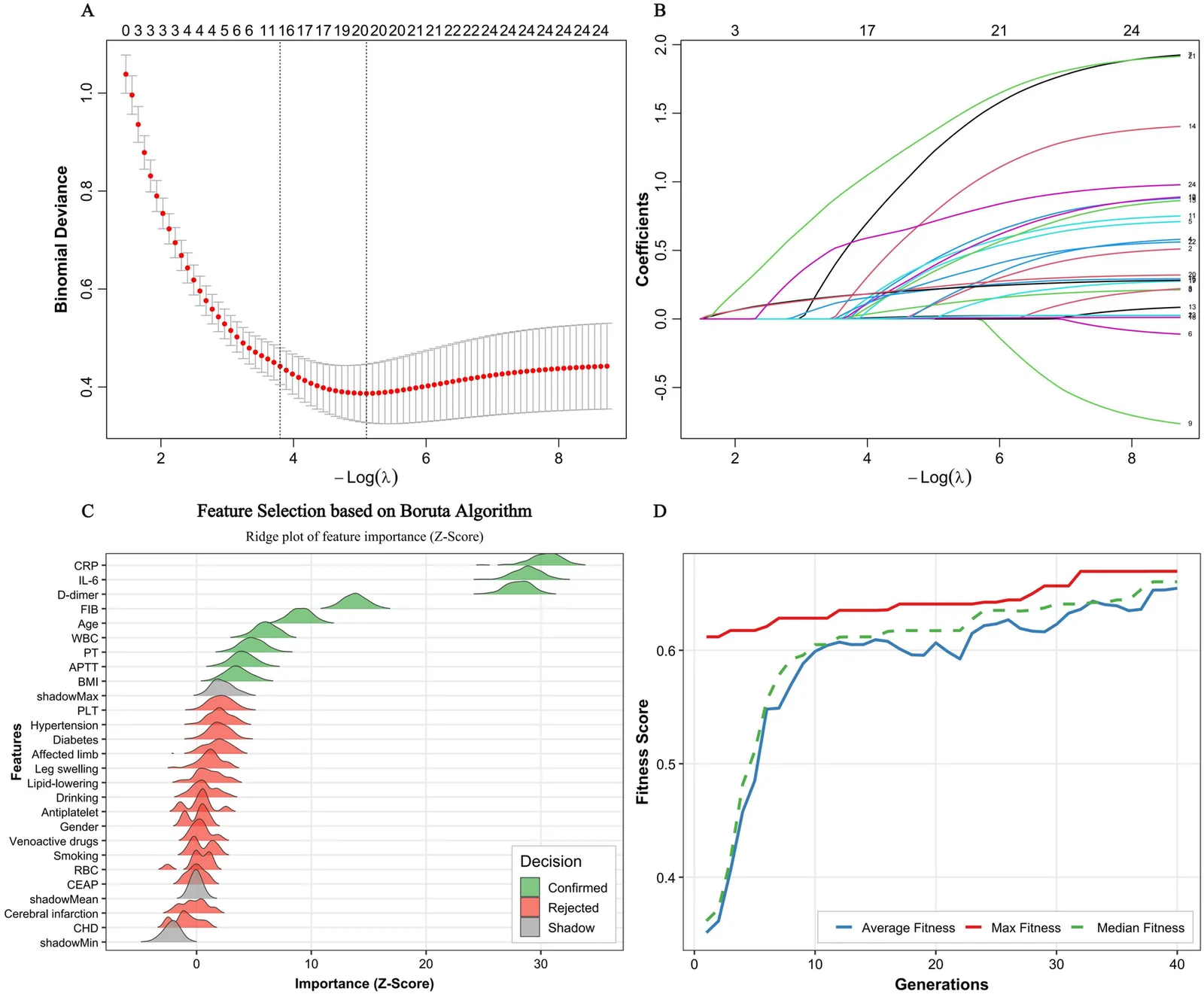
Feature-selection results using LASSO regression, the boruta algorithm, and a genetic algorithm. **(A)** Ten-fold cross-validation curve for LASSO logistic regression. Red points represent the mean cross-validated binomial deviance, and error bars indicate the corresponding standard errors. Vertical dotted lines indicate the cross-validation-derived penalty values; lambda.min was used to retain variables with nonzero coefficients. Numbers along the upper axis indicate the number of nonzero coefficients at each penalty value. **(B)** LASSO coefficient profiles of the 24 candidate variables across penalty values. **(C)** Ridge plot of variable importance derived from the Boruta algorithm. Confirmed variables are shown in green, rejected variables in red, and shadow variables in gray. **(D)** Evolution of the average, maximum, and median fitness scores across GA generations. Variables retained by all three methods were designated as consensus-selected variables; age, IL-6, CRP, D-dimer, and FIB constituted the three-method intersection. LASSO, least absolute shrinkage and selection operator; GA, genetic algorithm; IL-6, interleukin-6; CRP, C-reactive protein; FIB, fibrinogen; CEAP, Clinical-Etiology-Anatomy-Pathophysiology classification; WBC, white blood cell count; RBC, red blood cell count; PLT, platelet count; PT, prothrombin time; APTT, activated partial thromboplastin time; BMI, body mass index; CHD, coronary heart disease.

LASSO retained 19 of the 24 candidate variables at lambda.min, Boruta classified 9 variables as confirmed, and the original GA run selected 19 variables. The complete method-specific selection results are provided in Supplementary Table S2. The intersection of the three methods comprised age, IL-6, CRP, D-dimer, and FIB, which were designated as consensus-selected variables and entered into the full multivariable logistic regression model. Across 100 independent GA runs with different random seeds, the exact selected subsets showed some variability. Age, IL-6, CRP, D-dimer, and FIB were selected in all 100 runs, while selection frequencies for the remaining variables ranged from 20% to 100% (Supplementary Figure S1). These findings indicate consistent GA retention of the five consensus-selected variables, while also showing that the GA did not identify a unique invariant subset across runs. Feature-selection stability was further assessed across the 1,000 bootstrap resamples in which the complete three-method selection procedure was repeated *de novo*. Age, IL-6, CRP, D-dimer, and FIB were retained by the consensus procedure in 94.0%, 99.0%, 100.0%, 100.0%, and 97.0% of bootstrap resamples, respectively. Selection frequencies for the remaining candidate variables ranged from 52.0% to 75.0% (Supplementary Table S3). These results indicate that the five variables identified in the original cohort were frequently retained across bootstrap resamples, although other candidate variables were also selected in a proportion of resampled datasets.

Pairwise Spearman correlation coefficients among the five consensus-selected variables ranged from 0.01 to 0.29. The strongest correlation was observed between IL-6 and CRP (*ρ*=0.29). FIB showed modest correlations with IL-6 (*ρ*=0.22), CRP (*ρ*=0.21), D-dimer (*ρ*=0.16), and age (*ρ*=0.07). VIF values were 1.04 for age, 1.25 for IL-6, 1.28 for CRP, 1.23 for D-dimer, and 1.08 for FIB. All VIFs were substantially below 5, indicating no substantial multicollinearity among the selected variables (Supplementary Figure S2).

### Assessment of continuous-variable functional forms

Restricted cubic spline analyses showed no evidence of departure from linearity on the log-odds scale for age (*P* for nonlinearity=0.124), IL-6 (*P* for nonlinearity=0.106), CRP (*P* for nonlinearity=0.153), D-dimer (*P* for nonlinearity=0.130), or FIB (*P* for nonlinearity=0.673) (Supplementary Figure S3). These variables were therefore retained as linear terms on their original scales in the subsequent multivariable logistic regression models.

### Development and comparative evaluation of the nomogram

Four models were compared: the Caprini score alone (Model 1); age, IL-6, CRP, and D-dimer (Model 2); Model 2 plus FIB (Model 3); and Model 3 plus the Caprini score (Model 4). The AUCs were 0.722 (95% CI, 0.663–0.782), 0.837 (95% CI, 0.790–0.884), 0.844 (95% CI, 0.797–0.891), and 0.871 (95% CI, 0.830–0.912) for Models 1–4, respectively (Figure 3A). Compared with Model 1, Models 2 and 3 showed higher discrimination, with *Δ*AUCs of 0.115 (95% CI, 0.040–0.190; DeLong *P* = 0.003) and 0.122 (95% CI, 0.046–0.197; DeLong *P* = 0.002), respectively. Adding FIB to Model 2 resulted in a small AUC increase of 0.007 (95% CI, −0.004 to 0.017; DeLong *P* = 0.224). Models 2 and 3 showed similar optimism-corrected calibration and Brier scores, and their decision curves largely overlapped. Reclassification metrics are reported in Table 2 and were interpreted as exploratory. Given the limited incremental contribution of FIB, the reduced four-variable Model 2 was selected for nomogram presentation (Figure 3B). The complete regression parameters of the final four-variable Model 2 are presented in Supplementary Table S4. Using the original measurement scales, the linear predictor was calculated as LP = −11.74 + 0.25  ×  Age + 0.28 × IL-6 + 0.26 × CRP + 1.60 × D-dimer, where age was expressed in years, IL-6 in pg/mL, CRP in mg/L, and D-dimer in mg/L. The estimated probability of concurrent SVT at presentation was calculated as P = exp(LP)/[1 + exp(LP)]. The corresponding full five-variable nomogram for Model 3 is provided in Supplementary Figure S4 for comparison. Adding the Caprini score to Model 3 numerically increased the AUC from 0.844 to 0.871 (*Δ*AUC = 0.027; 95% CI, 0.000–0.055; DeLong *P* = 0.054). Reclassification metrics for the Model 4 versus Model 3 comparison are reported in Table 2 and were considered exploratory. Calibration was generally acceptable across all models, while decision curve analysis, used as the primary assessment of clinical net benefit, showed greater net benefit for Models 2–4 than for the Caprini-only model across most evaluated threshold probabilities (Figures 3C,D; Table 2).

**Figure 3:**
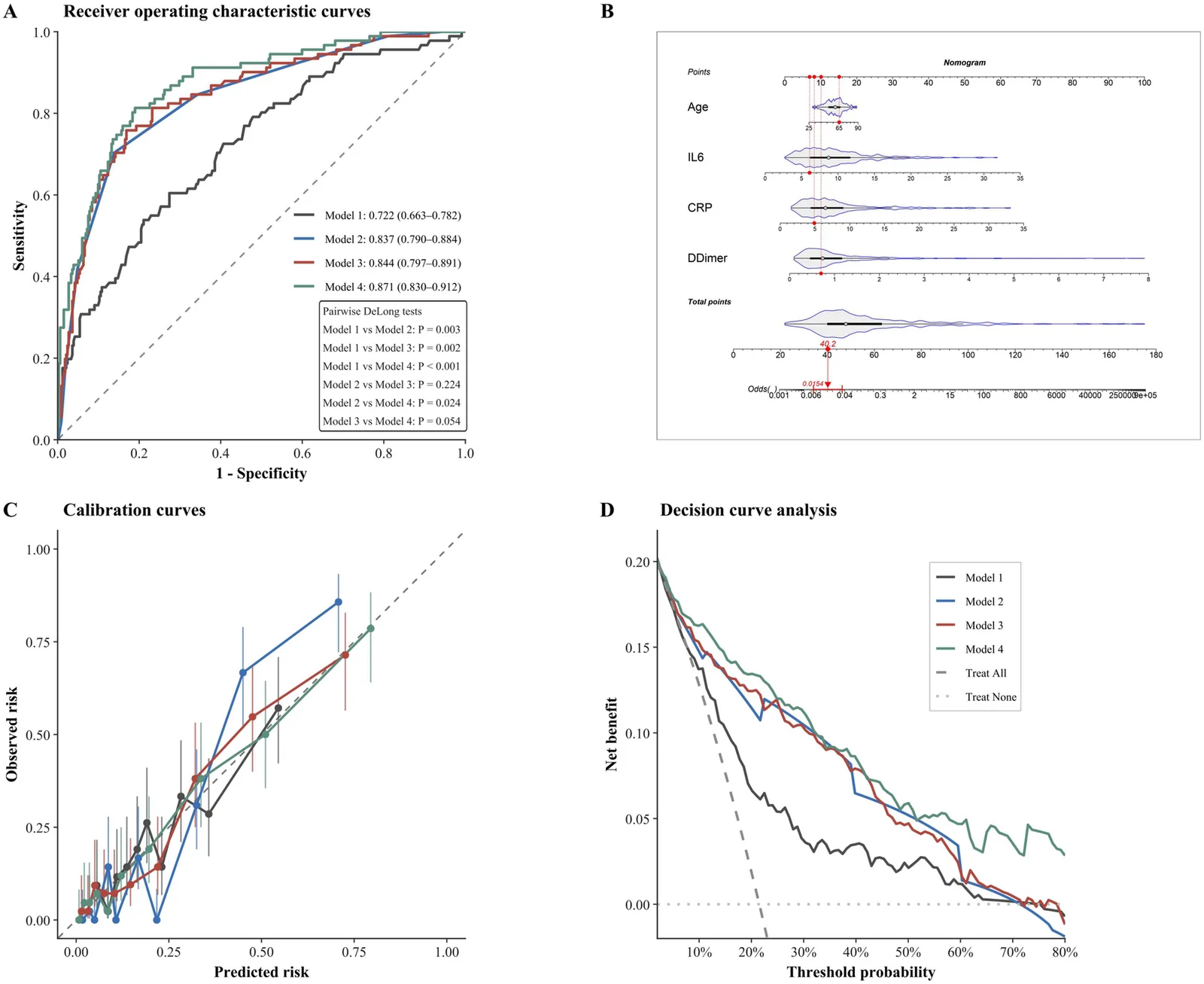
Development and comparative evaluation of the four models for estimating concurrent SVT. **(A)** Receiver operating characteristic curves comparing the Caprini score alone (Model 1), age, IL-6, CRP, and D-dimer (Model 2), Model 2 plus FIB (Model 3), and Model 3 plus the Caprini score (Model 4). AUCs with 95% confidence intervals are shown in the legend, and correlated AUCs were compared using DeLong's test. **(B)** Nomogram derived from Model 2 for individualized estimation of the probability of concurrent SVT at presentation. The total score is calculated by summing the points assigned to age, IL-6, CRP, and D-dimer and converting the total into an estimated probability of concurrent SVT. **(C)** Apparent calibration curves for Models 1-4 in the complete analytic cohort. The diagonal dashed line represents perfect agreement between predicted probabilities and observed proportions. **(D)** Decision curve analysis comparing the net benefit of Models 1-4 across a range of threshold probabilities. The “treat all” and “treat none” strategies are shown as reference curves. SVT, superficial vein thrombosis; AUC, area under the receiver operating characteristic curve; IL-6, interleukin-6; CRP, C-reactive protein; FIB, fibrinogen.

**Table 2:** Pairwise comparisons of incremental discrimination and reclassification performance among the four models for estimating concurrent SVT.

Model	NRI		IDI		Discrimination	
	Index (95% CI)	*P*	Index (95% CI)	*P*	*Δ*AUC (95% CI)	DeLong *P*
Model 2 vs. Model 1	0.582 (0.356–0.800)	<0.001	0.159 (0.092–0.223)	<0.001	0.115 (0.040–0.190)	0.003
Model 3 vs. Model 1	0.614 (0.398–0.836)	<0.001	0.169 (0.101–0.234)	<0.001	0.122 (0.046–0.197)	0.002
Model 3 vs. Model 2	0.251 (0.018–0.471)	0.033	0.011 (−0.002–0.023)	0.099	0.007 (−0.004–0.017)	0.224
Model 4 vs. Model 3	0.558 (0.332–0.770)	<0.001	0.071 (0.039–0.104)	<0.001	0.027 (0.000–0.055)	0.054

Model 1: Caprini score; Model 2: age + IL-6 + CRP + D-dimer; Model 3: age + IL-6 + CRP + D-dimer + FIB; Model 4: age + IL-6 + CRP + D-dimer + FIB + Caprini score. NRI and IDI represent the incremental performance of the first-listed model relative to the second-listed model. *Δ*AUC was calculated as the AUC of the first-listed model minus that of the second-listed model. The 95% confidence intervals for NRI, IDI, and *Δ*AUC were estimated using 500 paired bootstrap resamples, and correlated AUCs were compared using DeLong's test. Continuous NRI was considered exploratory because it captures changes in predicted probability regardless of their magnitude. Reclassification metrics were treated as secondary analyses and were not used as the primary basis for interpreting clinical net benefit.

NRI, net reclassification improvement; IDI, integrated discrimination improvement; AUC, area under the receiver operating characteristic curve; CI, confidence interval; IL-6, interleukin-6; CRP, C-reactive protein; FIB, fibrinogen.

### Internal validation and optimism-corrected performance

All 1,000 bootstrap iterations were successfully completed. The mean AUC optimism was −0.000287 for Model 1, 0.000019 for Model 2, 0.001697 for Model 3, and 0.003889 for Model 4, yielding optimism-corrected AUCs of 0.722384, 0.837131, 0.842091, and 0.867321, respectively. The near-zero optimism estimate for Model 2 therefore reflected a very small nonzero value rather than an exactly zero estimate. For the final four-variable Model 2, the apparent and optimism-corrected calibration intercepts were 0.000 and 0.001, respectively; the corresponding calibration slopes were 1.000 and 1.000, and the Brier scores were 0.120 and 0.121. Apparent and optimism-corrected performance metrics for all four models are provided in Supplementary Table S5. Overall, bootstrap correction produced only negligible changes in AUC for Models 1 and 2 and small reductions for Models 3 and 4, indicating limited estimated optimism within the study cohort.

### Multiple-imputation sensitivity analysis

Sensitivity analyses after multiple imputation yielded results consistent with the complete-case analysis. The AUCs for Models 1–4 were 0.718 (95% CI, 0.659–0.777), 0.839 (95% CI, 0.791–0.887), 0.847 (95% CI, 0.798–0.896), and 0.872 (95% CI, 0.831–0.913). The incremental contribution of FIB remained limited (Model 3 vs. Model 2: *Δ*AUC = 0.008; 95% CI, −0.003 to 0.019; DeLong *P* = 0.182), while adding the Caprini score to Model 3 produced a numerical AUC increase of 0.025 (95% CI, 0.000–0.050; DeLong *P* = 0.052). Calibration and decision-curve patterns were also similar to those observed in the complete-case analysis (Supplementary Table S6; Supplementary Figure S5).

## Discussion

This study developed and internally validated a multivariable logistic regression model for estimating the probability of concurrent SVT at presentation in patients with lower extremity varicose veins. A consensus feature-selection strategy integrating LASSO regression, the Boruta algorithm, and a genetic algorithm identified age, IL-6, CRP, D-dimer, and FIB as five consensus-selected variables. This intersection was used as a conservative modeling choice to favor parsimony and cross-method agreement rather than to define a uniquely established variable set. Repeated GA runs showed that all five variables were consistently retained despite some variation in the selected subsets. Adding FIB produced only a small improvement in model performance, so the four-variable model incorporating age, IL-6, CRP, and D-dimer was selected for nomogram presentation. This model showed higher discrimination than the Caprini score alone, while bootstrap validation indicated limited estimated optimism within the study cohort. Restricted cubic spline analyses showed no evidence of nonlinear log-odds relationships for the continuous variables within the observed data range. Although the three-method strategy was used to favor parsimony and cross-method agreement, the candidate-variable pool was evaluated in a cohort containing only 91 concurrent SVT events. Multi-stage feature selection may therefore remain sensitive to sampling variation. In the bootstrap stability analysis, age, IL-6, CRP, D-dimer, and FIB were retained in 94.0%–100.0% of resamples, supporting relatively frequent retention of these variables across resampled datasets. However, several other candidate variables were also selected in a substantial proportion of bootstrap samples, indicating that the selected variable set was not invariant. Residual selection instability and overfitting cannot be excluded despite internal validation, and the stability and transportability of the model require confirmation in independent cohorts.

The variables retained in the reduced model are consistent with biological processes involved in SVT. Venous thrombosis is generally related to the interaction of venous stasis, endothelial injury, and hypercoagulability (16, 17). In patients with varicose veins, chronic venous hypertension and impaired venous return may provide a local hemodynamic environment favorable to thrombus formation, while inflammatory and coagulation activation may further contribute to disease development (18, 19). Because IL-6, CRP, D-dimer, and FIB were measured during the same presentation period in which SVT status was ascertained, their temporal relationship with thrombosis cannot be determined in this study. Elevated biomarker levels may reflect an underlying inflammatory or coagulation state, a biological response to an existing thrombus, or both. Their inclusion in the model should therefore be interpreted as providing information associated with concurrent SVT status rather than as evidence that these biomarkers precede SVT or increase its future risk. IL-6 can promote tissue-factor expression, endothelial activation, and procoagulant responses, whereas CRP reflects systemic inflammatory activity and may also be associated with vascular endothelial dysfunction (20, 21). These biomarkers may therefore provide information relevant to concurrent SVT status, although the direction of the underlying biological relationship cannot be established from the present data. Increasing age is associated with changes in vascular structure, endothelial function, and coagulation balance (22, 23), while D-dimer reflects fibrin formation and degradation and is commonly used as an indicator of ongoing coagulation and fibrinolytic activity (24, 25). Together, age, IL-6, CRP, and D-dimer provide complementary demographic, inflammatory, and coagulation-related information for estimating concurrent SVT at presentation.

FIB was retained by all three feature-selection methods in the complete cohort, but its incremental contribution was limited when directly compared with the reduced four-variable model. FIB is both a coagulation substrate and an acute-phase reactant and may partly overlap with information captured by IL-6 and CRP (26, 27). Although the observed correlations and variance inflation factors did not indicate substantial multicollinearity, the addition of FIB increased the AUC by only 0.007, with no clear improvement in IDI, calibration, Brier score, or decision-curve performance. These findings support retaining FIB in the full data-driven model for methodological completeness while selecting the reduced model for nomogram presentation on the basis of parsimony. The reduced model may also be easier to implement when fibrinogen measurement is unavailable, although its transportability requires external validation.

The reduced model showed higher discrimination than the Caprini score alone in the present cohort. This difference should be interpreted in light of the distinct information captured by the two approaches and the timing of the study outcome. The Caprini score primarily summarizes pre-existing demographic and clinical risk factors and was developed for broad VTE risk assessment in hospitalized and perioperative populations, whereas the present model was developed specifically for concurrent SVT in patients with lower extremity varicose veins and incorporated biomarkers measured during the same clinical presentation. IL-6 and CRP may reflect contemporaneous inflammatory and endothelial activation, while D-dimer reflects ongoing fibrin formation and degradation. These biologically proximal signals may therefore help distinguish patients with and without an existing thrombotic process at presentation more effectively than a general clinical risk score. This interpretation is consistent with disease-specific VTE studies showing that general risk-assessment tools may have limited specificity in particular patient populations and that context-specific clinical and laboratory information can contribute to individualized thrombotic risk assessment (28, 29). Importantly, greater discrimination for concurrent SVT does not imply that IL-6, CRP, or D-dimer has a causal role or provides superior prediction of future thrombosis. Because these biomarkers were measured during the same presentation period in which SVT was identified, elevated concentrations may represent antecedent inflammatory or coagulation activity, a biological response to an already established thrombus, or both. The present data cannot distinguish among these possibilities. Thus, their contribution to the model should be interpreted as diagnostic association with concurrent SVT rather than evidence of temporal or causal effects.

Adding the Caprini score to the full five-variable model numerically increased the AUC from 0.844 to 0.871. The absolute AUC difference was 0.027 (95% CI, 0.000–0.055; DeLong *P* = 0.054). Because this comparison did not reach statistical significance, no additional discriminative or clinical value was inferred from adding the Caprini score. The nomogram provides an internally evaluated estimate of the probability of concurrent SVT at presentation based on four routinely available variables. However, because no independent external validation cohort was available, its transportability and real-world clinical utility remain unestablished. The model should not be used as a standalone tool for clinical decision-making before independent external validation. In particular, no validated probability cut-offs were established for triggering ultrasound surveillance, anticoagulation, or other interventions. The estimated probability generated by the nomogram should therefore not be used as a standalone basis for clinical decision-making. Clinical decisions should continue to incorporate symptoms, physical findings, ultrasonographic characteristics, bleeding risk, and clinician judgment.

Several limitations should be considered. First, this was a single-center retrospective study, and selection bias and residual confounding cannot be excluded. Although excluded patients were compared with the complete-case cohort, several baseline characteristics showed appreciable standardized differences, and the small number of excluded patients limited the precision of this assessment. In addition, center-specific referral patterns, diagnostic pathways, Doppler ultrasound practice, and laboratory testing procedures may have influenced the case mix and measured biomarker distributions. Although laboratory measurements were performed on the same hospital platform, differences in diagnostic and laboratory workflows across institutions may affect model calibration and transportability. Second, the model underwent bootstrap-based internal validation only, with a limited number of concurrent SVT events (*n* = 91), which may affect the precision and stability of multivariable estimates and leave residual risk of overfitting. Because Model 2 was evaluated as a fixed four-variable specification during bootstrap validation, its optimism estimate primarily reflects coefficient-estimation optimism and does not fully incorporate uncertainty arising from the preceding model-reduction step. Residual model-selection optimism therefore cannot be excluded. Although bootstrap resampling was used to assess both model optimism and feature-selection stability, internal resampling cannot substitute for independent external validation. Independent external validation in larger, multicenter cohorts is therefore required before clinical application. No validated probability thresholds were established for ultrasound surveillance, anticoagulation, or other interventions; therefore, nomogram estimates should not be used as standalone clinical decision criteria. Third, recent immobilization and quantitative measures of venous anatomical or hemodynamic severity, including great saphenous vein diameter, saphenofemoral junction reflux duration, and standardized hemodynamic measurements, were not consistently available and could not be included. Their absence may have limited representation of local venous disease severity and increased reliance on systemic inflammatory and coagulation biomarkers. Fourth, only baseline measurements were analyzed, without assessment of temporal changes in inflammatory or coagulation biomarkers. Finally, the inflammatory and coagulation biomarkers were measured during the same presentation period in which SVT status was ascertained. Temporal precedence therefore cannot be established, and elevated biomarker levels may partly reflect the biological response to an existing thrombus. Accordingly, these associations should not be interpreted as evidence that the biomarkers cause or predict future SVT. Prospective longitudinal studies with biomarker measurements preceding SVT occurrence are required to evaluate their potential role in future SVT risk.

## Conclusion

In summary, this study developed and internally validated a parsimonious nomogram incorporating age, IL-6, CRP, and D-dimer for estimating the probability of concurrent SVT at presentation in patients with lower extremity varicose veins. The four-variable model showed good discrimination and calibration and showed higher estimated net benefit within the evaluated threshold probabilities than the Caprini score alone within the study cohort. Because the model has not undergone independent external validation, validation in larger, multicenter cohorts is required before clinical application.

## Data Availability

The raw data supporting the conclusions of this article will be made available by the authors, without undue reservation.

## References

[1] Piazza G Krishnathasan D Hamade N Ujueta F Scimeca G Ortiz-Rios MD. Superficial vein thrombosis: a review. JAMA. (2025) 334(22):2020–30. 10.1001/jama.2025.15222 40952730

[2] Bettiol A Alibaz-Oner F Direskeneli H Hatemi G Saadoun D Seyahi E. Vascular behçet syndrome: from pathogenesis to treatment. Nat Rev Rheumatol. (2023) 19(2):111–26. 10.1038/s41584-022-00880-7 36544027

[3] Bailey AJM Luo OD Zhou SQ Wells PS. The incidence and risk of venous thromboembolism in patients with active malignancy and isolated superficial venous thrombosis: a systematic review and meta-analysis (the IROVAM-iSVT review). J Thromb Haemost. (2025) 23(6):1824–37. 10.1016/j.jtha.2025.03.019 40154793

[4] Gloviczki P Lawrence PF Wasan SM Meissner MH Almeida J Brown KR. The 2023 society for vascular surgery, American venous forum, and American vein and lymphatic society clinical practice guidelines for the management of varicose veins of the lower extremities. Part II: endorsed by the society of interventional radiology and the society for vascular medicine. J Vasc Surg Venous Lymphat Disord. (2024) 12(1):101670. 10.1016/j.jvsv.2023.08.011 37652254 PMC11523430

[5] Doruk S Karadeniz G Örün H Deniz S Polat G. Risk factors for short and long-term mortality in acute pulmonary thromboembolism: a retrospective study. Int J Gen Med. (2025) 18:5465–73. 10.2147/IJGM.S528501 40994882 PMC12455332

[6] Qiao L Yao Y Wu D Xu R Cai H Shen Y. The validation and modification of the caprini risk assessment model for evaluating venous thromboembolism after joint arthroplasty. Thromb Haemost. (2024) 124(3):223–35. 10.1055/a-2122-7780 37402391 PMC10890911

[7] Hayssen H Sahoo S Nguyen P Mayorga-Carlin M Siddiqui T Englum B. Ability of caprini and Padua risk-assessment models to predict venous thromboembolism in a nationwide veterans affairs study. J Vasc Surg Venous Lymphat Disord. (2024) 12(2):101693. 10.1016/j.jvsv.2023.101693 37838307 PMC10922503

[8] Pannucci CJ. Commentary on: an in-depth analysis of 1370 consecutive abdominoplasties: fascial plication increases risk of venous thromboembolism, and scoring for plication improves the caprini risk assessment model. Aesthet Surg J. (2024) 44(12):NP883–5. 10.1093/asj/sjae183 39163523

[9] Lobastov K Dubar E Schastlivtsev I Bargandzhiya A. A systematic review and meta-analysis for the association between duration of anticoagulation therapy and the risk of venous thromboembolism in patients with lower limb superficial venous thrombosis. J Vasc Surg Venous Lymphat Disord. (2024) 12(2):101726. 10.1016/j.jvsv.2023.101726 38008180 PMC11523472

[10] Van Royen FS Van Smeden M Van Doorn S Rutten FH Geersing GJ. Predictive factors of clot propagation in patients with superficial venous thrombosis towards deep venous thrombosis and pulmonary embolism: a systematic review and meta-analysis. BMJ Open. (2024) 14(4):e074818. 10.1136/bmjopen-2023-074818 38626964 PMC11029256

[11] Díez Vidal A Gómez López J Rivera Núñez MA Martínez Virto AM Tung Chen Y. Risk factors and complications associated with superficial venous thrombosis. Eur J Intern Med. (2024) 121:146–8. 10.1016/j.ejim.2023.12.001 38129196

[12] Cortese F Stolfi L Luzi G Tarsia G D’Addeo G De Francesco M. Superficial vein thrombosis: state of art. A review. Phlebology. (2025) 40(10):741–53. 10.1177/02683555251338747 40317195

[13] Zöller B Sundquist J Sundquist K Ohlsson H. The risk for venous thromboembolism and cardiometabolic disorders in offspring from thrombosis-prone pedigrees. J Thromb Haemost. (2024) 22(3):775–84. 10.1016/j.jtha.2023.11.024 38072377

[14] He L Luo L Hou X Liao D Liu R Ouyang C. Predicting venous thromboembolism in hospitalized trauma patients: a combination of the caprini score and data-driven machine learning model. BMC Emerg Med. (2021) 21(1):60. 10.1186/s12873-021-00447-x 33971809 PMC8111727

[15] Lurie F Passman M Meisner M Dalsing M Masuda E Welch H. The 2020 update of the CEAP classification system and reporting standards. J Vasc Surg Venous Lymphat Disord. (2020) 8(3):342–52. 10.1016/j.jvsv.2019.12.075 32113854

[16] Rabe E Hoffmann U Schimke A Heinken A Langer F Noppeney T. Determinants of late venous thromboembolic events after acute isolated superficial vein thrombosis in daily practice: 12 month results of the INSIGHTS-SVT study. Eur J Vasc Endovasc Surg. (2023) 66(5):697–704. 10.1016/j.ejvs.2023.08.031 37573936

[17] Ordieres-Ortega L Alonso-Beato R Pire-García T Moragón-Ledesma S López-Rubio M Lago-Rodríguez M-O. Superficial vein thrombosis in obese patients. J Clin Med. (2025) 14(14):5024. 10.3390/jcm14145024 40725717 PMC12295803

[18] Tang L Xu Y Wang L Pan J Wu Y. Development of a predictive nomogram model for early deep vein thrombosis in postoperative spontaneous intracerebral hemorrhage patients. Int J Gen Med. (2024) 17:4793–803. 10.2147/IJGM.S484611 39440100 PMC11495207

[19] Mathieu M-E Duffett L Caiano L Scarvelis D Code C Wells P. Management and outcomes of superficial vein thrombosis: a single-center retrospective study. Res Pract Thromb Haemost. (2024) 8(1):102263. 10.1016/j.rpth.2023.102263 38187826 PMC10770555

[20] Moderegger EL Dräger S Preuss SL Vorobyev A Terheyden P Kridin K. Increased risk of deep vein thrombosis, pulmonary embolism, and all-cause mortality in chronic venous disorder: a large-scale retrospective cohort study. Front Med (Lausanne. (2025) 12:1683970. 10.3389/fmed.2025.1683970 41133149 PMC12540070

[21] Liu L Wu Z. Superficial vein thrombophlebitis and deep vein thrombosis for BD patients with leg ulcers. Int J Low Extrem Wounds. (2023) 22(4):809–11. 10.1177/15347346211070669 34955056

[22] Debourdeau P Bertoletti L Font C López-Núñez JJ Gómez-Cuervo C Mahe I. Three-month outcomes in cancer patients with superficial or deep vein thrombosis in the lower limbs: results from the RIETE registry. Cancers (Basel. (2023) 15(7):2034. 10.3390/cancers15072034 37046695 PMC10093050

[23] Sun X-X Ling H Zhang L Chen R-B Zhong A- Feng L- Development and validation of a risk prediction model and prediction tools for post-thrombotic syndrome in patients with lower limb deep vein thrombosis. Int J Med Inform. (2024) 187:105468. 10.1016/j.ijmedinf.2024.105468 38703744

[24] Skeith L Malinowski AK El-Chaâr D Chan W-S Donnelly J Chauleur C. Low-dose aspirin versus placebo in postpartum venous thromboembolism: a multi-national, pilot, randomised, placebo-controlled trial. Lancet Haematol. (2025) 12(2):e109–19. 10.1016/S2352-3026(24)00338-7 39827892

[25] Amalia L Ardo RT. Clinical significance of D-dimer level and numeric rating scale with amount of sinus involvement in cerebral sinus thrombosis patients. Int J Gen Med. (2024) 17:2143–9. 10.2147/IJGM.S460407 38766601 PMC11102121

[26] Gwozdzinski L Pieniazek A Gwozdzinski K. The roles of oxidative stress and red blood cells in the pathology of the varicose vein. Int J Mol Sci. (2024) 25(24):13400. 10.3390/ijms252413400 39769165 PMC11678264

[27] Gianesini S De Luca L Feodor T Taha W Bozkurt K Lurie F. Cardiovascular insights for the appropriate management of chronic venous disease: a narrative review of implications for the use of venoactive drugs. Adv Ther. (2023) 40(12):5137–54. 10.1007/s12325-023-02657-0 37768506 PMC10611621

[28] Cui C Yin Q Long T Guan H Lao Z. Anticoagulation management in intracerebral hemorrhage patients with deep vein thrombosis: insights from unsupervised machine learning and nomogram analysis. Front Neurol. (2026) 16:1711123. 10.3389/fneur.2025.1711123 41561342 PMC12812592

[29] Chen W Cui C Lai C. Developing a predictive model for lower extremity deep vein thrombosis in acute ischemic stroke using a nomogram. Front Neurol. (2025) 16:1506959. 10.3389/fneur.2025.1506959 40589987 PMC12206819

