# Supplementary material for: Development and internal validation of a nomogram for estimating concurrent superficial vein thrombosis at presentation in patients with lower extremity varicose veins
Source: Front Med (Lausanne). 2026 Oct 2;13:1889714. doi: 10.3389/fmed.2026.1889714 (PMC13672201; doi:10.3389/fmed.2026.1889714)
Supplement: Supplementary file 1 [file Table1.docx]

| **Table S1** Descriptive comparison of available baseline characteristics between patients included in the complete-case cohort and those excluded because of missing biomarker data | | | | |
| --- | --- | --- | --- | --- |
| **Variables** | **Excluded**  **(n=8)** | **Included**  **(n=423)** | ***P*** | **Absolute SMD** |
| Age, years | 55.0 ± 8.6 | 59.2 ± 11.6 | 0.217 | 0.409 |
| BMI, kg/m² | 24.7 ± 2.3 | 25.4 ± 3.4 | 0.473 | 0.216 |
| WBC, ×10^9^/L | 7.40 ± 2.21 | 7.33 ± 1.50 | 0.933 | 0.036 |
| RBC, ×10^12^/L | 4.56 ± 0.54 | 4.50 ± 0.47 | 0.750 | 0.126 |
| PLT, ×10^9^/L | 258.4 ± 73.2 | 235.3 ± 48.2 | 0.403 | 0.373 |
| IL-6, pg/mL | 6.8 | 9.5 ± 4.7 | - | - |
| CRP, mg/L | 7.4 ± 4.0 | 7.8 ± 5.3 | 0.828 | 0.101 |
| D-dimer, mg/L | 0.49 ± 0.41 | 1.01 ± 0.94 | 0.153 | 0.716 |
| PT, s | 12.7 ± 1.5 | 11.9 ± 1.1 | 0.170 | 0.609 |
| APTT, s | 31.4 ± 4.1 | 30.9 ± 3.6 | 0.775 | 0.113 |
| FIB, g/L | 3.41 ± 0.30 | 3.39 ± 0.78 | 0.935 | 0.028 |
| Caprini score | 5.9 ± 2.0 | 5.4 ± 1.8 | 0.512 | 0.259 |
| SVT, n(%) | 3 (37.50) | 91 (21.51) | 0.380 | 0.356 |
| Male, n(%) | 3 (37.50) | 172 (40.66) | 1.000 | 0.065 |
| Smoking, n(%) | 2 (25.00) | 125 (29.55) | 1.000 | 0.102 |
| Drinking, n(%) | 4 (50.00) | 94 (22.22) | 0.083 | 0.604 |
| Hypertension, n(%) | 2 (25.00) | 161 (38.06) | 0.716 | 0.284 |
| Diabetes, n(%) | 0 (0.00) | 79 (18.68) | 0.361 | 0.678 |
| Coronary heart disease, n(%) | 0 (0.00) | 50 (11.82) | 0.605 | 0.518 |
| Cerebral infarction, n(%) | 1 (12.50) | 34 (8.04) | 0.495 | 0.147 |
| Antiplatelet drugs, n(%) | 4 (50.00) | 85 (20.09) | 0.061 | 0.660 |
| Lipid-lowering drugs, n(%) | 3 (37.50) | 120 (28.37) | 0.694 | 0.195 |
| Venoactive drugs, n(%) | 4 (50.00) | 192 (45.39) | 1.000 | 0.092 |
| Leg swelling, n(%) | 3 (37.50) | 176 (41.61) | 1.000 | 0.084 |
| CEAP classification, n(%) |  |  | 0.269 | 0.574 |
| C2-C3 | 7 (87.50) | 270 (63.83) |  |  |
| C4-C6 | 1 (12.50) | 153 (36.17) |  |  |
| Affected limb, n(%) |  |  | 0.052 | 0.686 |
| Unilateral | 4 (50.00) | 342 (80.85) |  |  |
| Bilateral | 4 (50.00) | 81 (19.15) |  |  |
| Absolute standardized mean differences were used to quantify the magnitude of between-group differences. Because only eight patients were excluded because of missing biomarker data, both *P* values and standardized mean differences should be interpreted cautiously. Comparisons of CRP, D-dimer, and FIB were based on the available observations in the excluded group and were considered exploratory. Because IL-6 was available for only one excluded patient, no formal between-group comparison or standardized mean difference was calculated. SVT, superficial vein thrombosis; CEAP, Clinical-Etiology-Anatomy-Pathophysiology; BMI, body mass index; WBC, white blood cell count; RBC, red blood cell count; PLT, platelet count; IL-6, interleukin-6; CRP, C-reactive protein; PT, prothrombin time; APTT, activated partial thromboplastin time; FIB, fibrinogen; SMD, standardized mean difference. | | | | |

| **Table S2** Complete variable-selection results from LASSO, Boruta, and the genetic algorithm | | | | |
| --- | --- | --- | --- | --- |
| **Variables** | **LASSO** | **Boruta** | **GA** | **Three-method intersection** |
| Age | Selected | Confirmed | Selected | Yes |
| Gender | Selected | Rejected | Selected | No |
| BMI | Selected | Confirmed | Not selected | No |
| Smoking | Selected | Rejected | Selected | No |
| Drinking | Selected | Rejected | Selected | No |
| Hypertension | Not selected | Rejected | Selected | No |
| Diabetes | Selected | Rejected | Selected | No |
| Coronary heart disease | Not selected | Rejected | Not selected | No |
| Cerebral infarction | Not selected | Rejected | Selected | No |
| Antiplatelet drugs | Selected | Rejected | Selected | No |
| Lipid-lowering drugs | Selected | Rejected | Selected | No |
| Venoactive drugs | Selected | Rejected | Selected | No |
| CEAP classification | Not selected | Rejected | Selected | No |
| Affected limb | Selected | Rejected | Selected | No |
| Leg swelling | Selected | Rejected | Selected | No |
| WBC | Selected | Confirmed | Not selected | No |
| RBC | Selected | Rejected | Not selected | No |
| PLT | Selected | Rejected | Selected | No |
| IL-6 | Selected | Confirmed | Selected | Yes |
| CRP | Selected | Confirmed | Selected | Yes |
| D-dimer | Selected | Confirmed | Selected | Yes |
| PT | Not selected | Confirmed | Selected | No |
| APTT | Selected | Confirmed | Not selected | No |
| FIB | Selected | Confirmed | Selected | Yes |
| LASSO selection was based on nonzero coefficients at lambda.min determined by 10-fold cross-validation. Boruta classified variables as confirmed or rejected according to their importance relative to shadow variables. GA results indicate whether each variable was included in the optimal subset obtained from the original genetic-algorithm run. The three-method intersection identifies variables retained by all three selection methods. The intersection was used as a conservative modeling rule to prioritize parsimony and cross-method agreement and should not be interpreted as an objectively established set of variables. LASSO, least absolute shrinkage and selection operator; GA, genetic algorithm; BMI, body mass index; CEAP, Clinical-Etiology-Anatomy-Pathophysiology; WBC, white blood cell count; RBC, red blood cell count; PLT, platelet count; IL-6, interleukin-6; CRP, C-reactive protein; PT, prothrombin time; APTT, activated partial thromboplastin time; FIB, fibrinogen. | | | | |

| **Table S3** Bootstrap selection frequencies of candidate variables across 1,000 resamples | | |
| --- | --- | --- |
| **Variables** | **Selected by three-method consensus, n** | **Selection frequency, %** |
| Age | 940 | 94.0 |
| Gender | 580 | 58.0 |
| BMI | 670 | 67.0 |
| Smoking | 520 | 52.0 |
| Drinking | 590 | 59.0 |
| Hypertension | 600 | 60.0 |
| Diabetes | 750 | 75.0 |
| Coronary heart disease | 530 | 53.0 |
| Cerebral infarction | 620 | 62.0 |
| Antiplatelet drugs | 560 | 56.0 |
| Lipid-lowering drugs | 530 | 53.0 |
| Venoactive drugs | 680 | 68.0 |
| CEAP classification | 540 | 54.0 |
| Affected limb | 560 | 56.0 |
| Leg swelling | 640 | 64.0 |
| WBC | 730 | 73.0 |
| RBC | 530 | 53.0 |
| PLT | 680 | 68.0 |
| IL-6 | 990 | 99.0 |
| CRP | 1,000 | 100.0 |
| D-dimer | 1,000 | 100.0 |
| PT | 590 | 59.0 |
| APTT | 580 | 58.0 |
| FIB | 970 | 97.0 |
| The complete feature-selection procedure, including LASSO, Boruta, genetic algorithm optimization, and three-method consensus selection, was repeated de novo in each of 1,000 bootstrap resamples. Selection frequency represents the proportion of bootstrap resamples in which each variable was retained by the three-method consensus. BMI, body mass index; CEAP, Clinical-Etiology-Anatomy-Pathophysiology; WBC, white blood cell count; RBC, red blood cell count; PLT, platelet count; IL-6, interleukin-6; CRP, C-reactive protein; PT, prothrombin time; APTT, activated partial thromboplastin time; FIB, fibrinogen. | | |

| **Table S4** Multivariable logistic regression parameters of the final four-variable model for estimating concurrent SVT at presentation | | | | | |
| --- | --- | --- | --- | --- | --- |
| **Variables** | **β coefficient** | **SE** | **Z** | ***P*** | **OR (95% CI)** |
| Intercept | -11.74 | 1.80 | -6.53 | <0.001 | - |
| Age, per 1 year | 0.25 | 0.04 | 5.82 | <0.001 | 1.28 (1.18-1.40) |
| IL-6, per 1 pg/mL | 0.28 | 0.05 | 6.03 | <0.001 | 1.32 (1.21-1.45) |
| CRP, per 1 mg/L | 0.26 | 0.04 | 6.55 | <0.001 | 1.30 (1.20-1.40) |
| D-dimer, per 1 mg/L | 1.60 | 0.28 | 5.68 | <0.001 | 4.94 (2.85-8.57) |
| Regression coefficients were estimated from the final multivariable logistic regression Model 2 using the original measurement scales. Odds ratios for continuous variables represent the change in odds per one-unit increase. SE, standard error; OR, odds ratio; CI, confidence interval; IL-6, interleukin-6; CRP, C-reactive protein; SVT, superficial vein thrombosis. | | | | | |

| **Table S5** Apparent and optimism-corrected performance of the four models for estimating concurrent SVT based on 1,000 bootstrap resamples | | | | | | | |
| --- | --- | --- | --- | --- | --- | --- | --- |
| **Model** | **Apparent AUC** | **Mean optimism** | **Optimism-corrected AUC** | **Calibration intercept, apparent/corrected** | **Calibration slope, apparent/corrected** | **Brier score, apparent/corrected** | **Successful/attempted bootstrap iterations** |
| Model 1 | 0.722097 | -0.000287 | 0.722384 | 0.000000/0.002402 | 1.000000/1.019228 | 0.146985/0.148237 | 1,000/1,000 |
| Model 2 | 0.837151 | 0.000019 | 0.837131 | 0.000000/0.001280 | 1.000000/1.000459 | 0.119788/0.121069 | 1,000/1,000 |
| Model 3 | 0.843787 | 0.001697 | 0.842091 | 0.000000/0.000578 | 1.000000/0.985957 | 0.118364/0.120410 | 1,000/1,000 |
| Model 4 | 0.871210 | 0.003889 | 0.867321 | 0.000000/0.001014 | 1.000000/0.972796 | 0.106997/0.109871 | 1,000/1,000 |
| Model 1: Caprini score; Model 2: age + IL-6 + CRP + D-dimer; Model 3: age + IL-6 + CRP + D-dimer + FIB; Model 4: age + IL-6 + CRP + D-dimer + FIB + Caprini score. Apparent performance was estimated in the complete analytic cohort. For the full data-driven model, the complete feature-selection procedure was repeated de novo within each bootstrap resample; models with fixed specifications were refitted as described in the Methods. Optimism was estimated by comparing performance in each bootstrap sample with that obtained when the corresponding bootstrap-developed model was applied to the original cohort. Optimism-corrected performance was calculated by subtracting the mean optimism from the apparent estimate. All 1,000 bootstrap iterations were successfully completed. AUC, area under the receiver operating characteristic curve. | | | | | | | |

| **Table S6** Sensitivity analysis of discrimination and reclassification performance after multiple imputation | | | | | | |
| --- | --- | --- | --- | --- | --- | --- |
| **Model** | **NRI** | | **IDI** | | **Discrimination** | |
|  | **Index (95% CI)** | ***P*** | **Index (95% CI)** | ***P*** | **ΔAUC (95% CI)** | **DeLong *P*** |
| Model 2 vs Model 1 | 0.600 (0.365-0.814) | <0.001 | 0.167 (0.102-0.232) | <0.001 | 0.121 (0.045-0.197) | 0.002 |
| Model 3 vs Model 1 | 0.663 (0.442-0.866) | <0.001 | 0.177 (0.110-0.243) | <0.001 | 0.129 (0.052-0.206) | <0.001 |
| Model 3 vs Model 2 | 0.268 (0.033-0.492) | 0.018 | 0.010 (-0.003-0.022) | 0.120 | 0.008 (-0.003-0.019) | 0.182 |
| Model 4 vs Model 3 | 0.542 (0.327-0.761) | <0.001 | 0.065 (0.033-0.097) | <0.001 | 0.025 (0.000-0.050) | 0.052 |
| Sensitivity analysis was performed in the full clinically eligible cohort (n=431) after multiple imputation by chained equations for missing IL-6, CRP, D-dimer, and FIB values. Twenty imputed datasets were generated using predictive mean matching with 20 iterations and five donors. The predefined model specifications were refitted without repeating feature selection. Model 1: Caprini score; Model 2: age + IL-6 + CRP + D-dimer; Model 3: age + IL-6 + CRP + D-dimer + FIB; Model 4: age + IL-6 + CRP + D-dimer + FIB + Caprini score. NRI and IDI represent the incremental performance of the first-listed model relative to the second-listed model. ΔAUC was calculated as the AUC of the first-listed model minus that of the second-listed model. The 95% confidence intervals for NRI, IDI, and ΔAUC were estimated using 500 paired bootstrap resamples, and correlated AUCs were compared using DeLong’s test. Continuous NRI was considered exploratory. NRI, net reclassification improvement; IDI, integrated discrimination improvement; AUC, area under the receiver operating characteristic curve; CI, confidence interval; IL-6, interleukin-6; CRP, C-reactive protein; FIB, fibrinogen. | | | | | | |

**Fig. S1** Variable selection frequencies across 100 independent genetic algorithm runs. The complete GA procedure was repeated 100 times using different random seeds while all tuning parameters were kept unchanged. Bars represent the proportion of runs in which each candidate variable was included in the selected subset. Age, IL-6, CRP, D-dimer, and FIB, which constituted the three-method consensus set, were selected in all 100 runs. GA, genetic algorithm; IL-6, interleukin-6; CRP, C-reactive protein; FIB, fibrinogen.

**Fig. S2** Correlation structure and multicollinearity assessment among the five consensus-selected variables. Spearman correlation matrix among age, interleukin-6 (IL-6), C-reactive protein (CRP), D-dimer, and fibrinogen (FIB). Values within the cells represent Spearman correlation coefficients (ρ), and color intensity indicates the direction and magnitude of the correlations. Variance inflation factors (VIFs) are shown for the five consensus-selected variables. All VIF values were below 5, indicating no substantial multicollinearity among the selected variables. IL-6, interleukin-6; CRP, C-reactive protein; FIB, fibrinogen; VIF, variance inflation factor.

**Fig. S3** Restricted cubic spline assessment of the functional forms of continuous variables for concurrent superficial vein thrombosis. Multivariable restricted cubic spline logistic regression with three knots was used to examine the functional forms of (A) age, (B) IL-6, (C) CRP, (D) D-dimer, and (E) FIB in relation to concurrent SVT at presentation. Each analysis was adjusted for the remaining variables in the corresponding multivariable model. Solid lines represent adjusted odds ratios, shaded areas represent 95% confidence intervals, and the vertical cyan dashed lines indicate the median values of the corresponding variables, which were used as reference values (OR=1.0). P values for overall association and nonlinearity are shown in each panel. No variable showed evidence of departure from linearity on the log-odds scale. SVT, superficial vein thrombosis; IL-6, interleukin-6; CRP, C-reactive protein; FIB, fibrinogen.

**Fig. S4** Full five-variable nomogram for estimating concurrent superficial vein thrombosis in patients with lower extremity varicose veins. The nomogram was derived from the full data-driven Model 3 incorporating age, interleukin-6 (IL-6), C-reactive protein (CRP), D-dimer, and fibrinogen (FIB). For each variable, the corresponding points are determined from the upper points scale and summed to obtain the total score, which is then converted into the estimated probability of concurrent SVT. SVT, superficial vein thrombosis; IL-6, interleukin-6; CRP, C-reactive protein; FIB, fibrinogen.

**Fig. S5** Multiple-imputation sensitivity analysis of the four models for estimating concurrent SVT. Sensitivity analyses were performed in the full clinically eligible cohort (n=431) after multiple imputation of missing biomarker values. (A) Receiver operating characteristic curves for Models 1-4. The corresponding AUCs with 95% confidence intervals and pairwise DeLong test results are shown. (B) Nomogram derived from the reduced four-variable Model 2 incorporating age, IL-6, CRP, and D-dimer. (C) Calibration curves comparing predicted probabilities and observed proportions of concurrent SVT for Models 1-4. The diagonal dashed line represents perfect agreement. (D) Decision curve analysis comparing the net benefit of Models 1-4 across threshold probabilities; the “treat all” and “treat none” strategies are shown as reference curves. Model 1: Caprini score; Model 2: age + IL-6 + CRP + D-dimer; Model 3: age + IL-6 + CRP + D-dimer + FIB; Model 4: age + IL-6 + CRP + D-dimer + FIB + Caprini score. SVT, superficial vein thrombosis; AUC, area under the receiver operating characteristic curve; IL-6, interleukin-6; CRP, C-reactive protein; FIB, fibrinogen.
